# Low Childhood Vaccination Coverage among Ukrainian Refugees in Norway. A Nationwide, Register-Based Cohort Study, 2022–2023

**DOI:** 10.1007/s10903-025-01725-7

**Published:** 2025-07-16

**Authors:** Evy Therese Dvergsdal, Suzanne Campbell, Nora Dotterud Lerstad, Margrethe Greve-Isdahl, Angela Susan Labberton, Bo Terning Hansen, Hinta Meijerink

**Affiliations:** Norwegian Institute of Public Health, Oslo, Norway

**Keywords:** Vaccination coverage, Child immunisation programmes, Refugees, Migration health, Norway, Ukraine

## Abstract

Low vaccination coverage is highlighted as a challenge in Ukraine and among Ukrainian refugees, however there is limited knowledge on coverage among Ukrainian refugees living in European countries. Individual-level data from the National Population Register for children (<20 years) with at least one parent from Ukraine were linked to the Norwegian Immunisation Registry (SYSVAK). We estimated vaccination coverage according to Norwegian Childhood Immunisation Program recommendations among 1) Ukrainian refugee children (i.e. immigrated after 01.02.2022) (N = 8,804) and 2) children born in Norway to Ukrainian refugee parent(s) (N = 224), compared to national coverage. National coverage rates were 95.8% to 96.5% (2-year-olds), 94.5% to 96.7% (9-year-olds), and 90.0% to 94.0% (16-year-olds). Corresponding rates among Ukrainian refugee children were 39.5% to 60.5%, 27.3% to 58.6%, and 9.2% to 34.3%, respectively. Coverage among Norwegian-born children of Ukrainian refugees was >91%. In all, 31% of Ukrainian refugee children had no vaccinations registered in SYSVAK. Coverage for Human Papilloma Virus (HPV) catch-up vaccination among Ukrainian refugee boys was lowest (9%), however, routine HPV vaccination coverage was 63%. Ukrainian children with a refugee background showed lower vaccination coverage than the national coverage. We found large differences between routine versus catch-up vaccination. Our findings suggest that access to catch-up vaccination, routines for retrospective registration of vaccines given outside of Norway and surveillance reporting, as well as vaccine hesitancy should all be considered when assessing childhood vaccination coverage among Ukrainian refugees. These factors should be addressed to improve vaccine uptake and surveillance in this population.

**Supplementary Information:**

The online version contains supplementary material available at 10.1007/s10903-025-01725-7.

## Introduction

Since the full-scale invasion of Ukraine in 2022, over 80,000 Ukrainians have applied for collective protection in Norway, where approximately one-third are children. Persons with Ukrainian background now represent the second largest immigrant group in Norway, accounting for over 1.5% of the population aged 0–20 years [1].

Insufficient vaccination coverage among Ukrainian children has been raised as an area of concern previously [2, 3]. This concern also applies to some other immigrant groups in Norway [4, 5]. Therefore, in 2024, the Norwegian Childhood Immunisation Programme (NCIP) analysed coverage statistics by parental country of birth for the first time. Compared to national rates (range 93–97%), children with Ukrainian parents showed particularly low coverage (range 25–51%) [6]. The NCIP estimates the vaccine coverage for all children at age 2, 9 and 16 years [6, 7].

There are likely multiple reasons for the low coverage [2, 3, 8]. This study aims to analyse coverage among Ukrainian refugee children in Norway and investigate the extent to which discrepancies reflect low vaccination uptake versus other challenges such as system barriers, inadequate routines in follow-up and stringent coverage definitions.

## Methods

### Study Context

The NCIP offers free and voluntary vaccination against 13 diseases to children and adolescents up to age 20 years. Ukrainian refugees typically spend their first time in Norway at an asylum reception centre, where an initial assessment of vaccination status is recommended. However, only vaccines against polio and measles are recommended to be prioritized in this setting.

Further follow-up of children aged 0–5 years takes place at municipal child health clinics, while older children are followed up through the school health services. Municipal child health clinics base appointment invitation on regularly updated information on inhabitants from the National Population Register (NPR). Registration of vaccinations in the Norwegian Immunisation Registry (SYSVAK) is mandatory and has very high completeness [7]. Retrospective registration of vaccinations administered outside Norway should be done routinely. Written documentation is required for vaccination against measles, mumps, and rubella (MMR) administered abroad, while assessment of prior immunisation for other vaccines rely on the healthcare providers’ discretion. Catch-up vaccination should always be offered if vaccination status is unclear.

### Participants

**Fig. 1:**
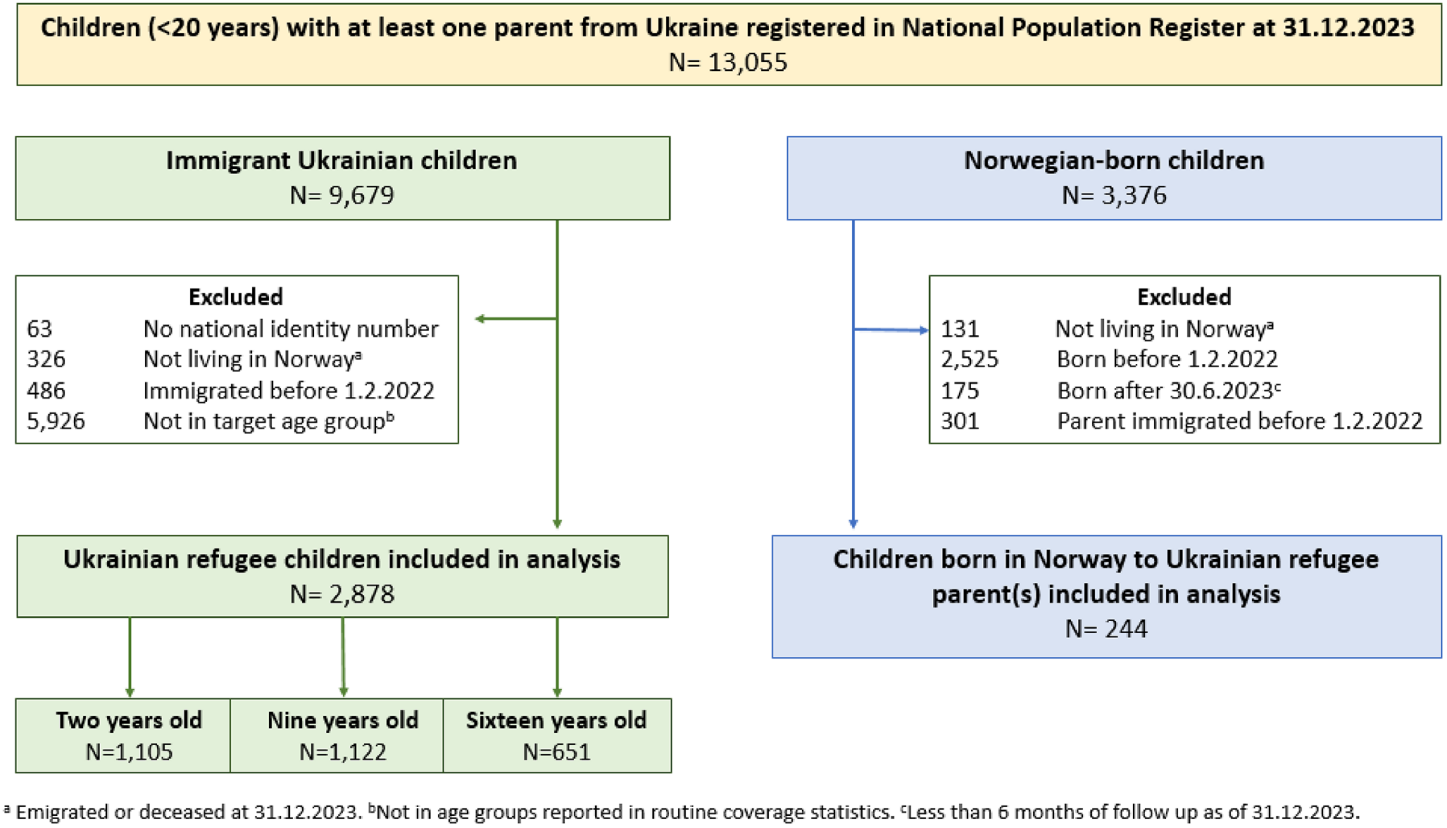
Flow chart showing the selection of the study population for analyses reported in this study, split by Ukrainian children immigrated to Norway (green) and those born in Norway to recently immigrated Ukrainian parents (blue)

In this retrospective cohort study, we identified all children and adolescents (< 20 years) with at least one parent from Ukraine (*N* = 13,055) as recorded in NPR at 31.12.2023. We included (1) Ukrainian refugee children (i.e. immigrated after 01.02.2022 and born in Ukraine) (*N* = 8,804), and (2) Children born in Norway to Ukrainian refugee parent(s) (*N* = 224) (Fig. 1). For group 1), we included those aged 2, 9, and 16 years during the study period to estimate vaccine coverage as per NCIP reporting. The three chosen ages allow surveillance of completed infant vaccinations at 2 years, school-entry vaccinations at 9 years and end of lower secondary school at 16 years [4, 6, 7]. For group 2), we included only children born before 1 July 2023 to allow a minimum of 6 months follow-up time for vaccination. 

### Data Collection and Measures

Individual-level data from the NPR (age, sex, immigration date, and birth country of the child and parents) were linked using the unique national identification number to SYSVAK (vaccine-preventable disease, vaccination and registration date). Vaccines recommended in Ukraine and Norway are shown in Supplementary Table 1 (Online Resource 1).

### Analyses

Vaccination coverage was defined as the proportion of children who received the recommended vaccine doses against the specified disease for each age group. Results are given as absolute numbers and proportions with 95% confidence intervals (95%CI). To provide context for the vaccination coverage among children with Ukrainian background, we also calculated the corresponding coverage rates among all children in Norway aged 2, 9 or 16 years by 31.12.2023. For Human Papilloma Virus (HPV) vaccination, which is not part of the immunisation programme in Ukraine, we performed a sub-analysis among Ukrainian refugee children attending Norwegian school during the age that the HPV vaccine is routinely offered (12-year-olds) – to capture the difference between catch-up and routine vaccination.

## Results

We included 2878 Ukrainian refugee children and 244 Norwegian-born children to Ukrainian parent(s) in analyses (Fig. 1). Further characteristics of the study population is shown in Table 1.

**Table 1:** Characteristics of the study population

		Immigrant Ukrainian children^a^			Norwegian-born children to Ukrainian parent(s)^b^
		2-year-olds	9-year-olds	16-year-olds	
		N(%)	N(%)	N(%)	N(%)
Total Ukrainian background		1 105	1 122	651	244
Sex	Female	516 (46.7)	547 (48.8)	293 (45.0)	123 (50.4)
	Male	589 (53.3)	575 (51.3)	358 (55.0)	121 (49.6)
Country of birth	Norway	n.a	n.a	n.a	244 (100)
	Ukraine	1 083 (98.0)	1 119 (99.7)	646 (99.2)	n.a
	Other / unknown	22 (2.0)	3 (0.3)	5 (0.8)	n.a
Parents’ country of birth	Ukrainian mother	124 (11.2)	101 (9.0)	65 (10.0)	57 (23.4)
	Ukrainian father	52 (4.7)	66 (5.9)	45 (6.9)	22 (9.0)
	Both Ukrainian	929 (84.1)	955 (85.1)	541 (83.1)	165 (67.6)

^a^Immigrated after 1 February 2022. ^b^ Born before 1 July 2023 to allow a minimum of 6 months follow-up time. n.a = not applicable

### Vaccination Coverage among Ukrainian Refugee Children

National vaccination coverage rates across all vaccines ranged from 95.8 to 96.5% for 2-year-olds, 94.5–96.7% for 9-year-olds, and 90.0–94.0% for 16-year-olds (Table 2). In contrast, the corresponding ranges among Ukrainian refugee children were 39.5–60.7%, 27.3–58.6%, and 9.2–34.3%. Using a less stringent coverage definition (at least one dose), these rates among the Ukrainian refugee children were 60.1–70.2%, 58.7–65.5%, and 14.8–47.2%. Almost one-third (31%, 882) of Ukrainian refugee children included in this study did not have any vaccinations registered in SYSVAK.

HPV vaccination coverage among 16-year-old Ukrainian refugees represents catch-up vaccination in Norway and was especially low at 9.2%. The sub-analysis among Ukrainian refugee children attending Norwegian school during the school year that the HPV vaccine is routinely offered (12-year-olds) showed substantially higher coverage; 70.3% (girls) and 64.2% (boys). In comparison, national coverage for HPV among 12-year-olds was 94% (girls) and 92% (boys).

**Table 2:** The proportion of children with registered vaccinations, among Ukrainian refugee children (immigrated to Norway after 1 February 2022 with at least one parent from Ukraine) between 1 February 2022-31 December 2023, and the national coverages, between 1 January 2023-31 December 2023

	Vaccine coverage					
	Fully vaccinated NCIP^a^ Ukrainian children		At least one dose^b^ Ukrainian children		Fully vaccinated NCIP National coverage	
	%	95%CI	%	95%CI	%	95%CI
**2-year-olds**						
Hepatitis B	39.5	36.6–42.4	70.2	67.4–72.9	95.8	95.7–96.0
Measles, mumps, and rubella	59.0	56.0-61.9	60.1	57.1–63.0	95.8	95.6–95.9
Diphtheria and tetanus	60.7	57.8–63.6	69.9	67.1–72.6	96.5	96.3–96.6
Pertussis	60.5	57.6–63.4	69.9	67.1–72.6	96.5	96.3–96.6
Poliomyelitis	60.3	57.3–63.2	70.1	67.3–72.8	96.5	96.3–96.6
Haemophilus influenzae type b	51.8	48.8–54.7	66.2	63.4–69.0	96.3	96.2–96.5
All vaccines above	15.4	13.3–17.6	26.1	23.5–28.8	94.2	94.0-94.4
**9-year-olds**						
Hepatitis B	27.3	36.6–42.4	61.1	58.2–64.0	n.a^c^	n.a
Measles, mumps, and rubella	58.6	56.0-61.9	58.7	55.8–61.6	96.7	96.6–96.9
Diphtheria and tetanus	55.6	57.8–63.6	65.3	62.5–68.1	94.8	94.7–95.0
Pertussis	36.6	57.6–63.4	65.0	62.1–67.8	94.5	94.3–94.6
Poliomyelitis	52.3	57.3–63.2	65.5	62.6–68.3	94.7	94.5–94.8
All vaccines above	17.3	15.1–19.6	53.2	50.2–56.2	91.2	91.0-91.4
**16-year-olds**						
Hepatitis B	19.8	16.8–23.1	41.6	37.8–45.5	n.a^c^	n.a
Measles, mumps, and rubella	34.3	30.6–38.0	39.2	35.4–43.0	94.0	93.8–94.2
Diphtheria and tetanus	23.7	20.4–27.1	46.9	43.0-50.8	92.5	92.3–92.7
Pertussis	19.8	16.8–23.1	46.7	42.8–50.6	92.3	92.1–92.5
Poliomyelitis	31.8	28.2–35.5	47.2	43.3–51.1	92.5	92.3–92.7
Human papillomavirus						
Female	11.3	7.9–15.5	16.0	11.3–18.9	92.3	92.0-92.6
Male	9.2	6.4–12.7	14.8	12.0-20.8	90.0	89.6–90.2
All vaccines above	3.7	2.4–5.4	11.5	9.2–14.2	82.2	82.0-82.5

^a^Fully vaccinated according to the Norwegian Childhood Immunisation Programme (NCIP) using the algorithm that takes into account number of recommended vaccine doses with correct interval.^b^At least one dose of the recommended vaccines given after minimum age. See Supplementary methods (Online Resource 1) for further details of calculation of coverages.^c^Universal hepatitis B vaccination was included in the NCIP by introduction of a hexavalent infant vaccine in 2016 and was previously only provided for selected risk groups. The birth cohorts included for 9- and 16-year-olds in this analysis have not been offered Hepatitis B-vaccine in the NCIP. CI = confidence interval. n.a = not applicable

### Vaccination Coverage among Norwegian-born Children with Ukrainian Refugee Parents

Among Norwegian-born children with Ukrainian refugee parents, the coverage of at least one dose of rotavirus vaccine, diphtheria-tetanus-pertussis-poliomyelitis-haemophilus influenzae type b-hepatitis B vaccine (DTaP-IPV-Hib-HepB), and pneumococcal vaccine were 93.4%, 91.8% and 92.2% respectively (Table 3).

**Table 3:** The proportion of children registered with at least one dose of the vaccines recommended in the first year of life according to the Norwegian childhood immunisation programme (NCIP), among Norwegian-born children to Ukrainian refugee parent(s) and all Norwegian-born children, between 1 February 2022-31 December 2023

		At least 1 vaccine dose			
	Timing	Norwegian-born children to Ukrainian refugee parent(s) (*N* = 244)		All Norwegian-born children (*N* = 73,068)	
		%	95%CI	%	95%CI
Rotavirus	6 weeks	93.4	89.6–96.2	96.5	96.4–96.7
Diphtheria, tetanus, pertussis, Poliomyelitis, Haemophilus influenzae type b and hepatitis B	3, 5 and 12 months	91.8	87.6–94.9	98.0	97.9–98.1
Pneumococcal disease	3, 5 and 12 months	92.2	88.1–95.2	98.0	97.9–98.1
Any vaccines		93.9	90.1–96.5	98.3	98.2–98.4

CI = confidence interval

## Discussion

Although standard NCIP coverage statistics at ages 2, 9 and 16 years indicate that vaccination coverage in the Ukrainian refugee population is far below national estimates, this gap almost disappears when the analysis is limited to infants born in Norway to Ukrainian refugees. Similarly, HPV vaccination coverage increases substantially when adolescent refugees eligible for routine vaccination are considered separately. These two findings of substantially higher coverage in the context of routine vaccination suggest that vaccine hesitancy, may not be the main contributor for low vaccine uptake registered among Ukrainian refugees in Norway. Low childhood vaccination rates in Ukraine have been documented previously, with multiple contributors including supply issues and vaccine hesitancy [2, 3, 8].

We observed very low HPV catch-up vaccination coverage among 16-year-old Ukrainian refugees. Considering the much higher coverage observed among those eligible for routine HPV vaccination, it seems unlikely that the low rate among the 16-year-olds can be largely attributed to vaccine hesitancy. Moreover, since HPV vaccination is not part of the Ukrainian programme, there is no issue of missing retrospective registration in Norway, and the observed coverage reflects real uptake. We suggest that delayed catch-up vaccination, or no offer, in the NCIP may contribute to lower coverage in this population. Insufficient provider recommendations have also been cited as a barrier to immunization among immigrants in other countries [9]. Compared to pre-school catch-up vaccination, which takes place at child health clinics with parents, school-based catch-up may face organizational barriers leading to untimeliness or no offer at all. For recently arrived children to Norway, standard NCIP coverage statistics will mainly reflect catch-up vaccinations given in Norway as well as retrospective registration of vaccinations given abroad. Our results suggest that both practices can be improved.

Lack of retrospective registration of vaccinations administered in Ukraine may contribute to the low vaccination coverage observed in Norway, as the rates observed are even lower than those reported in Ukraine [10] for all cohorts in the study population. Missing documentation of previous vaccinations from Ukraine is likely a challenge for healthcare providers responsible for retrospective registration. Lastly, the NCIP uses stringent vaccination coverage definitions requiring very close compliance with NCIP recommendations (see Supplementary methods, Online resource 1 for more details). Counting children with at least one dose moderately increased coverage for all vaccines except MMR. However, all estimates remained insufficient across all age groups.

Among the children born in Norway to Ukrainian parents, vaccination coverage was close to the national average. This might indicate that many Ukrainian parents are positive to vaccination, at least when they and their children are integrated into the preventive health care system from an early age and receive timely information about vaccinations. However, a gap remains compared to national rates for all cohorts in the study population, suggesting that hesitancy may still play a role.

Refugees and other newly arrived immigrants face multiple potential barriers in accessing healthcare - including preventative immunisations – such as language, lack of information, confidence/trust, and logistical barriers [11]. It is therefore of utmost importance that the host country has robust systems and routines to ensure vaccines are offered in a timely and appropriate manner.

### New Contribution to the Literature

To our knowledge this is the first study that has investigated vaccination coverage among Ukrainian refugees in Europe using nationwide data. Our findings provide an important contribution and have significant implications for policymakers in the field of public and immigrant health and access to immunisation programmes.

## Conclusion

Low childhood vaccination coverage among Ukrainian refugees in Norway appears multifactorial. Our results indicate that vaccine hesitancy may not be the primary explanation, since the coverage was much higher in routine vaccination settings than in non-routine settings. Organisational factors, such as how and to what extent catch-up vaccinations are offered, how coverage is defined, and the extent of registration of prior vaccinations in Ukraine, likely contribute to the discrepancies from national estimates. Standard coverage statistics should thus be interpreted with caution. Specifically for HPV, catch-up vaccination should be ensured for adolescents arriving after the routine vaccination age at 12 years. Further insight into the reasons behind under-vaccination of immigrant children in Norway is warranted.

## Electronic Supplementary Material

Below is the link to the electronic supplementary material.

Supplementary Material 1

## Data Availability

No datasets were generated or analysed during the current study.
