# Supplementary material for: Low Childhood Vaccination Coverage among Ukrainian Refugees in Norway. A Nationwide, Register-Based Cohort Study, 2022–2023
Source: J Immigr Minor Health. 2025 Jul 16;27(6):1129–34. doi: 10.1007/s10903-025-01725-7 (PMC12669341; doi:10.1007/s10903-025-01725-7)
Supplement: Supplementary file 1 — Supplementary Material 1 [file 10903_2025_1725_MOESM1_ESM.docx]

## Supplementary Information

### **Supplementary methods**

### **Description of the Childhood Immunisation Programmes in Norway and Ukraine**

In Norway, child health centres and school health services provide information on and vaccines in the Norwegian Childhood Immunisation Programme (NCIP) to all children and adolescents up to 20 years of age and who have temporary or permanent residency. The vaccines offered provide protection against thirteen different diseases (Supplementary Table 1). The first vaccine in the NCIP is administered at six weeks old, and the last vaccine is administered at 12 years old (10th grade). If for some reason the child has not received one or more of the vaccines in the programme when it is usually offered, they are entitled to receive any of the vaccines included in the NCIP until they turn 20 years old*.* Vaccination is voluntary and free of charge [1]. All children arriving in Norway from other countries should be offered vaccinations according to the NCIP, tailored to address any gaps in their immunity based on their prior vaccine coverage. Children from Ukraine require varying vaccination schedules, depending on their age at the time of arrival, their previous vaccination history, and the intervals between the vaccines they have received.

The vaccination schedule in Ukraine covers ten diseases (Supplementary Table 1). There are some differences in the type og vaccines offered and the vaccine schedule between the Norwegian and Ukrainian immunization programs [2]. In Norway children are offered rotavirus vaccine, pneumococcal vaccine, and HPV vaccine, none of which are included in the Ukrainian immunization program. Moreover, Ukraine uses whole-cell pertussis vaccines, whereas Norway uses acellular pertussis vaccines, both combined with diphtheria and tetanus (DTwP). Whole-cell pertussis vaccines confer broader and more prolonged protection compared to acellular vaccines [3]. This is reflected in the Ukrainian schedule, where primary vaccination comprises four doses and no subsequent booster dose [2]. In Ukraine, children receive the first dose of the measles, mumps, rubella (MMR) vaccine at 12 months and the second dose at 6 years. In Norway, the first dose is given at 15 months and the second dose at 11 years [4].

According to the World Health Organization, the official vaccination coverage in Ukraine for the first dose of the DTP-containing vaccine was 92.5% and 77.8% in 2023 and 2022, respectively. The coverage for the third dose of the DTP-containing vaccine was 83.4% and 72.9% in 2023 and 2022, respectively. For the first dose of the measles-containing vaccine, coverage was 92.4% and 74.1% in 2023 and 2022, respectively, while for the second dose, coverage was 87.3% and 69.1% in 2023 and 2022, respectively [4].

*Supplementary Table 1: Overview of the childhood vaccination programmes in Norway and Ukraine.*

| **Age** | **Vaccination schedule Norway** | **Vaccination schedule Ukraine** |
| --- | --- | --- |
| Day one |  | 1^st^ Hepatitis B |
| 3-5 days |  | Bacille Calmette Guérin (BCG) |
| 6 weeks | 1^st^ rotavirus  Bacille Calmette Guérin (BCG)* |  |
| 2 months |  | 1^st^ diphtheria, tetanus (DT)  1^st^ pertussis  1^st^ poliomyelitis  1^st^ Haemophilus influenzae type B (Hib)  2^nd^ hepatitis B |
| 3 months | 2^nd^ rotavirus  1^st^ diphtheria, tetanus, pertussis, poliomyelitis (DTP-IPV)  1^st^ hepatitis B  1^st^ Hib  1^st^ pneumococcal disease |  |
| 4 months |  | 2^nd^ DT  2^nd^ pertussis  2^nd^ poliomyelitis  2^nd^ Hib |
| 5 months | 2^nd^ DTP-IPV  2^nd^ hepatitis B  2^nd^ Hib  2^nd^ pneumococcal disease |  |
| 6 months |  | 3^rd^ DT  3^rd^ pertussis  3^rd^ poliomyelitis  3^rd^ hepatitis B |
| 12 months | 3^rd^ DTP-IPV  3^rd^ hepatitis B  3^rd^ Hib  3^rd^ pneumococcal disease | 1^st^ Measles, mumps, rubella (MMR)  3^rd^ Hib |
| 15 months | 1^st^ MMR |  |
| 18 months |  | 4^th^ DT  4^th^ pertussis  4^th^ poliomyelitis |
| 6 years |  | 5^th^ DT  5^th^ poliomyelitis  2^nd^ MMR |
| 7 years | 4^th^ DTP-IPV |  |
| 11 years | 2^nd^ MMR |  |
| 12 years | 1^st^ , 2^nd^ and 3^rd^ human papillomavirus (HPV) |  |
| 14 years |  | 6^th^ Poliomyelitis |
| 16 years | 5^th^ Dtp-IPV | 6^th^ DT |

** In Norway, BCG vaccination is specifically recommended for certain groups of children. It is advised for all children coming from Ukraine or those with at least one parent from Ukraine, as Ukraine experiences a notable prevalence of tuberculosis [5].*

### **The National Immunisation Registry SYSVAK and estimation of vaccination coverage**

SYSVAK is the national, electronic immunisation registry that records an individual’s vaccination status and is used to estimate vaccination coverage in Norway. SYSVAK was established in 1995 and is one of the national health registries. The registry is authorised in the Health Register Act and associated SYSVAK register regulations.

For the NCIP, coverage statistics are presented for all vaccines included in the program. The coverage statistics are produced for different age groups and indicate the percentage of children in a cohort who have been fully vaccinated at a given age. The coverage statistics are produced at the municipal level, county level and national level [6].

To estimate vaccination coverage in Norway, SYSVAK uses a built-in algorithm to calculate coverage for the different birth cohorts. This gives a snapshot of the actual protection against the diseases included in the Childhood Immunisation Programme. The algorithm rules are based on the recommendations and immunisation schedule in the programme. To be considered fully protected, a person needs to have received the vaccine at recommended age and with recommended intervals between doses. There are also alternative rules for immunisation schedules (e.g. children who followed a vaccination schedule in another country before residing in Norway) [7].

Vaccines administered within the time range and age limitations accepted in the Summary of Product Characteristics (SmPC) are counted as valid doses in SYSVAK. The algorithm is evaluated on a regular basis and follows the rules below for any of the vaccination schemes in NCIP:

• the minimum age at which the first dose can be counted as a valid dose (Supplementary Table 2),

• the minimum age at which a later dose can be counted as a valid dose,

• the minimum interval between a new dose and the previous dose, in order for the new dose to be counted as valid, and

• the period of a vaccination’s validity with respect to the date at which it is administered (i.e. the period of protection offered by the dose).

It is a complex system developed over many years and allows adjustments to improve accuracy in calculating coverage, identify unvaccinated individuals and hence improve vaccination coverage over time.

*Supplementary Table 2: Minimum age for vaccines in the Norwegian Childhood Immunisation programme.*

| **Vaccine** | **Minimum age of first dose** | **Minimum age of last dose** |
| --- | --- | --- |
| Rotavirus | 6 weeks | n.a. |
| Diphtheria and tetanus | 6 weeks | n.a. |
| Pertussis | 6 weeks | n.a. |
| Polio | 6 weeks | n.a. |
| Hepatitis B | n.a. | n.a. |
| Hib | 6 weeks | 11 months |
| Pneumococcal disease | 6 weeks | 11 months |
| MMR | 12 months | 3 years |
| HPV | 9 years | n.a. |

*n.a. = not applicable*

## Supplementary References

1. HelseNorge. The Childhood Immunisation Programme Oslo2024 [Available from: https://www.helsenorge.no/en/vaksiner-og-vaksinasjon/the-childhood-immunisation-programme/.

2. WHO Immunization Data Portal - All Data [Internet]. 2024 [cited 16 September 2024]. Available from: https://immunizationdata.who.int/global?topic=Vaccination-schedule&location=.

3. Chen Z, He Q. Immune persistence after pertussis vaccination. Hum Vaccin Immunother. 2017;13(4):744-56.

4. Bentdal YE BJ, Bergsaker MR, Bruun T, Dvergsdal E, Einhaug A, Feiring B, Fenne O, Greve-Isdahl M, Grosvold I, Johansen T, Knudsen CV, Mengshoel AT, Nilsen ØJ, Nordstrand K, Olsen E, Rykkvin R, Stålcrantz J, Trogstad L, Watle SV, Wester AL, Wiklund BS, Østlie I, Aase A. Barnevaksinasjonsprogrammet i Norge. Rapport for 2023. Oslo: Folkehelseinstituttet; 2024.

5. Folkehelseinstituttet. Tuberkulosevaksine (BCG-vaksine) -håndbok for helsepersonell 2024 [Available from: https://www.fhi.no/va/vaksinasjonshandboka/vaksiner-mot-de-enkelte-sykdommene/tuberkulosevaksinasjon/?term=#indikasjoner.

6. SYSVAK Nv. About SYSVAK 2023 [Available from: https://www.fhi.no/en/va/norwegian-immunisation-registry-sysvak/about-sysvak/).

7. Hagerup-Jenssen M, Kongsrud S, Riise Ø R. Suboptimal MMR2 vaccine coverage in six counties in Norway detected through the national immunisation registry, April 2014 to April 2017. Euro Surveill. 2017;22(17).

## List of abbreviations

| BCG | Bacille Calmette Guérin |
| --- | --- |
| DT | Diphtheria Tetanus |
| DTP-IPV | Diphtheria Tetanus Pertussis Poliomyelitis |
| DTaP-IPV-Hib-HepB | Diphtheria Tetanus Pertussis Poliomyelitis Haemophilus influenzae type b Hepatitis B |
| Hib | Haemophilus influenzae type b |
| MMR | Measles Mumps Rubella |
| NCIP | Norwegian Childhood Immunisation Programme |
| NPR | National Population Register |
| SYSVAK | Norwegian Immunisation Registry |
